# Persistent fibrosis, hypertrophy and sarcomere disorganisation after endoscopy-guided heart resection in adult *Xenopus*

**DOI:** 10.1371/journal.pone.0173418

**Published:** 2017-03-09

**Authors:** Lindsey Marshall, Céline Vivien, Fabrice Girardot, Louise Péricard, Barbara A. Demeneix, Laurent Coen, Norin Chai

**Affiliations:** 1 Evolution des Régulations Endocriniennes, Département Régulations, Développement et Diversité Moléculaire, UMR CNRS 7221, Muséum National d'Histoire Naturelle, Sorbonne Université, Paris, France; 2 Ménagerie du Jardin des Plantes, Muséum National d’Histoire Naturelle, Paris, France; University of California Irvine, UNITED STATES

## Abstract

Models of cardiac repair are needed to understand mechanisms underlying failure to regenerate in human cardiac tissue. Such studies are currently dominated by the use of zebrafish and mice. Remarkably, it is between these two evolutionary separated species that the adult cardiac regenerative capacity is thought to be lost, but causes of this difference remain largely unknown. Amphibians, evolutionary positioned between these two models, are of particular interest to help fill this lack of knowledge. We thus developed an endoscopy-based resection method to explore the consequences of cardiac injury in adult *Xenopus laevis*. This method allowed in situ live heart observation, standardised tissue amputation size and reproducibility. During the first week following amputation, gene expression of cell proliferation markers remained unchanged, whereas those relating to sarcomere organisation decreased and markers of inflammation, fibrosis and hypertrophy increased. One-month post-amputation, fibrosis and hypertrophy were evident at the injury site, persisting through 11 months. Moreover, cardiomyocyte sarcomere organisation deteriorated early following amputation, and was not completely recovered as far as 11 months later. We conclude that the adult *Xenopus* heart is unable to regenerate, displaying cellular and molecular marks of scarring. Our work suggests that, contrary to urodeles and teleosts, with the exception of medaka, adult anurans share a cardiac injury outcome similar to adult mammals. This observation is at odds with current hypotheses that link loss of cardiac regenerative capacity with acquisition of homeothermy.

## Introduction

Ischemic heart disease is the leading cause of mortality worldwide [1]. Myocardial infarction results in the loss of cardiomyocytes. In adult humans, these cardiomyocytes cannot be replaced. Organ function is maintained through adaptive tissue remodelling, including ventricular hypertrophy, often leading to heart failure [2,3]. Fundamental research using model organisms focuses increasingly on deciphering mechanisms and factors of repair and scarring, with the hope to unlock the ability to restore damaged cardiac tissue in humans [4]. Two main model organisms used in cardiac regenerative studies are zebrafish and mice [4,5]. Both show marked differences, adult zebrafish having a highly efficient cardiac regenerative capacity [6,7] while adult mice lack this ability [8,9].

Amphibians, such as anurans (i.e. frogs) and urodeles (i.e. salamanders), are evolutionarily placed between these two organisms and have been historical models for heart development and cardiovascular physiology [10–13]. Like zebrafish, urodeles such as the neotenic axolotl [14,15] and adult eastern newt [16–19], can regenerate their heart ventricle. Although *Xenopus* has been considered as a powerful model organism for regeneration research over the past decades [20], only limited information is available on the outcome of cardiac injury in this species and other frogs.

The idea to explore heart damage in frogs was first reported in 1875 although the outcome was not clear [21,22]. Nearly 100 years later, frog myocardial regeneration studies were resumed. Rumyantsev stated that the adult frog heart could partially regenerate based on the observation of “apparently newly formed myocardial tissue […] within the scar”, as well as mitotic cardiomyocytes and disorganised or dedifferentiated myofibres [22–24]. Thereafter the exact extent of cardiac regeneration in anurans remained inconclusive, requiring more thorough analysis. It is surprising that heart regeneration has not been revisited in the *Xenopus* model since the regenerative capacity of tail, limb, retina and nerve tissue has been well described and continues to be explored [20,25]. We decided to reconsider *Xenopus* as a model for studying heart injury. Other authors have recently underlined the interest in investigating cardiac regeneration in this model [26].

Currently four approaches are used for inducing cardiac damage: i) genetic ablation of cardiomyocytes, ii) myocardial infarction induction by left anterior descending coronary artery ligation, iii) tissue removal by ventricular resection and iv) tissue damage by cryoinjury (freezing), cautery injury (burning) or mechanical injury (squeezing or electric shock) [26–29]. Difficulties of the documented methods include exteriorising the heart, observation of extremely small vessels, reproducibility of the extent of damage and lack of injury visualisation. Such technical complexities could explain conflicting cardiac regeneration results observed in mice [8,9,30–32].

Aiming to overcome these difficulties yet maintain accuracy, we developed and applied an endoscopy-guided approach for ventricular resection as a new, alternative cardiac injury technique. Endoscopy has numerous benefits such as live internal visualisation, standardised biopsy size, high precision and therefore increased reproducibility. Clinically, the main advantages include less stress for the animal and internal organs, ability to accurately collect samples for a definitive diagnosis, precise prognosis and direct therapy [33].

Using the endoscopy-guided cardiac injury procedure we showed that the adult *Xenopus* heart was unable to regenerate. In the first week after injury, genes associated with proliferation were not affected, while genes linked to sarcomere structure were down regulated and those related to inflammation, fibrosis and hypertrophy were up regulated. At the tissue level, fibrous scarring, cardiomyocyte hypertrophy and sarcomere disorganisation were evident near the injury site, persisting 11 months post-amputation. Thus, adult *Xenopus* can complement adult mammalian models to study the consequences of heart injury after tissue removal. Furthermore, adapting our minimally invasive method of cardiac injury to other species could help limit surgical stress-related issues.

## Materials and methods

### Animals and ethics statement

The care and treatment of animals were in accordance with Institutional and National Guidelines (Commission de Génie Génétique, “Direction Départementale des Services Vétérinaires”, European Union Directive 2010/63/EU, agreement decision No. C75-05-01-2 for the European Convention for vertebrate animals used for experimental and other scientific purposes). All protocols used in this study were approved under the reference number 68–037 by the Ethical Committee of the National Museum of Natural History (Paris). Animals were purchased from the *Xenopus* Biological Resource Centre (Rennes, France).

Studies involved adult *Xenopus laevis* (>5-year-old), only females to limit the experimental variations, housed in a facility with 12:12 hours light:dark cycles, 18–20 °C ambient temperature, dechlorinated and filtered water and a commercial diet (TetraRubin® granules).

### Equipment

The procedures were performed with an endoscope system (Karl Storz) consisting of a 2.7 mm diameter, 18 cm length, 30 ° oblique rigid telescope with a 4.8 mm operating sheath, an endovideo camera and monitor, xenon light source and light cable and 1.7 mm endoscopic biopsy forceps. Carbon dioxide (CO_2_) insufflator with silicone tubing was used for insufflation.

### Analgesia and anaesthesia

For a better internal visualisation, frogs were fasted 24 hours prior to anaesthesia. Pre-surgical preparations included hydration of the animal in a shallow dechlorinated water water-bath. Analgesia was performed with an initial dose of butorphanol (1 mg/kg; Torbugésic Vet®; Zoétis France) followed by meloxicam (0.4 mg/kg; Metacam®; Boehringer-Ingelheim), both injected into the lymph sacs. After ten minutes, anaesthesia was performed by transferring the animals to a bath of buffered 1% tricaine methanesulfonate (Ethyl 3-aminobenzoate methanesulfonate; Sigma) for 8 minutes. Loss of righting reflex suggested a light stage of anaesthesia. A surgical plane was indicated by the loss of withdrawal reflexes.

### Endoscopy procedures

With the animal positioned in dorsal recumbence, the surgical field was aseptically prepared using a sterile gauze moistened with a dilute povidone-iodine solution (Betadine® solution 10%, Méda Pharma), left for 10–15 seconds on the surgical site.

A 3 mm paramedian skin incision was made just beneath the sternum. Care was taken to not damage the mid-ventral vein. Following skin incision the abdominal membrane was elevated, incised and carefully dissected. The telescope-sheath system was inserted into the pleuroperitoneal cavity, which was insufflated (S1A Fig and S1 File). Typically, a CO_2_ insufflation pressure of 0.5mm Hg with a flow rate not exceeding 0.5 l/min was used.

After endoscope insertion, the heart was located behind the falciform ligament (S1B Fig and S1 File), then using the endoscopic biopsy forceps the falciform ligament was opened and the single ventricle chamber was immediately apparent (S1C Fig and S1 File). The pericardial sac was carefully pinched open (S1D Fig and S1 File) and then during the ventricle diastole, an amputation was performed towards the heart apex (S1E and S1F Fig and S1 File). Once the scope was removed, the animal deflated immediately, naturally removing the CO_2_. The coelomic membrane and the skin were closed in one layer using interrupted sutures with monofilament nylon (S1 File). The animal was then transferred to an anaesthetic-free bath and rinsed copiously with fresh, well-oxygenated dechlorinated water. Operated frogs were fasted 24 hours after operation. Animals were subjected to the full amputation procedure (AMP), anesthetised and fasted but not operated (CTRL) or, for qPCR analysis, the procedure was stopped after opening the pericardium, leaving the ventricle intact (SHAM).

After surgery, the animals were monitored to full recovery and sacrificed (“experimental sacrifice”) for heart collection at 1, 3 and 7 dpa (days post-amputation), or 1, 2, 3, 6 and 11 mpa (months post-amputation). Animals that showed signs of ill health were not used in the study and were sacrificed (“ethical sacrifice”).

### Sample preparation

For section labelling, each adult *Xenopus* heart was collected, paraformaldehyde-fixed (4% in phosphate buffered saline (PBS; Sigma) for 20 hours at 4 °C), equilibrated overnight in 15% sucrose in PBS, mounted in FSC22® frozen section compound (Sigma), and sagittal sections were obtained (8 μm-thickness) at -24°C using a cryostat (Leica CM30050S).

### Immunohistochemistry

Sagittal heart sections were pre-incubated with 1% sodium dodecyl sulfate (Sigma) in PBS for 5 minutes to allow for antigen unmasking. Then immunodetection was carried out as previously described [34], using the following antibodies: mouse anti-cardiac tropomyosin (CH1 1:1000; DSHB), rabbit anti-fibronectin (fn 1:500; F3648, Sigma), rabbit anti-atrial natriuretic factor (ANF known in *Xenopus* as, and referred to in the manuscript as NPPA 1:300; AB5490, Millipore), and the appropriate secondary antibodies (1:1500; Invitrogen). For cardiomyocyte cell membranes staining with wheat germ agglutinin (WGA) AlexaFluor® 594 conjugate (Invitrogen), sections were washed three times in PBS, incubated for 10 minutes with WGA (5 μg.ml^-1^ in PBS), then washed again three times in PBS and once in PBS-T (0.1% Tween-20 (Sigma) in PBS) buffer.

All sections were counterstained with DAPI (4′,6-diamidino-2-phenylindole; Molecular Probes) for 10 minutes, followed by mounting with Prolong® gold antifade reagent (Molecular Probe) and imaged under confocal microscope (Leica TCS SP5) or a fluorescence microscope (Leica DM5500B microscope and DFC450C camera with Prior Lumen200 light source). All fluorescent images were post-treated in Photoshop (Adobe), and merged using NIH ImageJ software [35]. To better visualise differences in WGA intensity, the signal was transformed by converting red colour images to “Red/Green”-scale using ImageJ/Image/Lookup Tables.

### Picrosirius red (PSR) labelling

Sagittal heart sections were post-fixed for 30 minutes in formalin 10% (Sigma), followed by overnight fixation in Bouin’s solution (Sigma), and then extensively washed with tap water. Sections were incubated for one hour in PSR solution: 0.1% direct red 80 (Sigma) and 0.1% fast green FCF (Sigma) in saturated aqueous picric acid (1.2% picric acid in water; Sigma). Sections were quickly washed 10 times in distilled water, twice in 70% ethanol, then in 100% ethanol (2x15 minutes) and finally in Safesolv solution (Q Path®, VWR International; 2x15 minutes), before being mounted in safemount medium (Q Path®, VWR International). The slides were then imaged using a stereomicroscope (Leica MZ16F with QImaging Retiga camera) or a light microscope (Leica DM5500B and DFC450C camera).

### Cell area quantification

The cross-sectional area of cardiomyocytes was measured using images displaying orthogonal views of WGA-labelled cardiomyocytes, to analyse for concentric hypertrophy. In Photoshop (Adobe) cell outlines were traced in red using a Wacom stylus pen and graphic tablet, then individual cell areas filled with blue before the outlines were deleted, and the image was saved as TIFF. Images were opened in NIH ImageJ software [35], and using the “Analyze Particles” setup, the filled areas were automatically calculated for each cell. Count was performed on 2 or 3 independent heart sections, corresponding to minima of 300 cell areas for each group. Area size of <10 pixels was excluded. Box and whisker (min to max) plots with tukey whiskers removed were shown and an unpaired non-parametric t-test (Mann Whitney) was performed to compare the groups with the control.

### Gene monitoring by Real-time quantitative PCR

For RT-qPCR, independent biological replicates were collected from CTRL, SHAM and AMP animals. Hearts were collected, cleaned from atrium and arterial bulb (only the ventricle part was kept), then cut in half and thoroughly washed in PBS to eliminate the blood (which might compete with heart tissue gene expression as blood cells are nucleated), before being snap frozen in liquid nitrogen. Total RNAs were extracted from snap-frozen adult *Xenopus* heart ventricles using RNAqueous® Phenol-free total RNA isolation kit (Ambion), then quantified and quality controlled using Qubit (Thermo Fisher Scientific) and 2100 Bioanalyzer (Agilent Technologies) respectively. Samples with RNA integrity (RIN) of ≥7 (average RIN 8.6) were reverse transcribed using High-Capacity cDNA Reverse Transcription kit (Applied Biosystems) using a MyCyclerTM (Bio-Rad), following manufacturers recommendations. RT-qPCR reactions were performed in duplicate for each sample using Power SYBR® master mix on a QuantStudioTM6 Flex Real-Time PCR System (Applied Biosystems), following manufacturer recommendations. Primers (MWG Biotech) were designed using Primer Blast [36] and the relevant sequences are listed in S1 Table. Ct data were collected using ExpressionSuite Software (Applied Biosystems) and analysed using Excel (Microsoft). The Cts for each technical duplicate were averaged and normalised (ΔCt) against the geometric mean of two reference genes (*smarcd1* and *smn2*), which were chosen after analysis by the NormFinder software [37]. These normalisers were chosen among 12 putative normalisers, already known to be expressed in adult *Xenopus laevis* cardiac ventricles (*actnl6a*, *actn3*, *akt1*, *cebpb*, *col1a1*, *cxcl8*, *fn1*, *hif1a*, *smarcd1*, *tert*; our unpublished data) or to be good normalisers in other tissues (*smn2*, *odc*); they were ranked on the basis of their expression stability in the samples presented in this paper and the best pair was retained. Variations of expression were quantified by the ΔΔCts method [38], using the SHAM condition as references for each experimental time-points and fold changes were computed as 2^-ΔΔCt^. Statistical analyses were performed on ΔCts with Prism 7 (GraphPad). Outliers were removed with ROUT test method (Q = 1%). One-Way ANOVA followed by Holm-Sidak’s post-test were performed for datasets that passed a Shapiro-Wilk normality test; Kruskal-Wallis non-parametric test followed with Dunn's post-tests were performed for those that did not pass this test (namely *fn1*, 3 dpa; *col1a1*, 7 dpa; *nppb*, 7dpa; *actn3*, 3 dpa; *tnnt2*, 1 dpa; *cxcl8*, 7 dpa). *, p < 0.05, **, p < 0.01, ***, p < 0.001, ****, p < 0.0001).

## Results

### Application of endoscopic biopsy as a ventricular resection method in *Xenopus*

**Fig 1:**
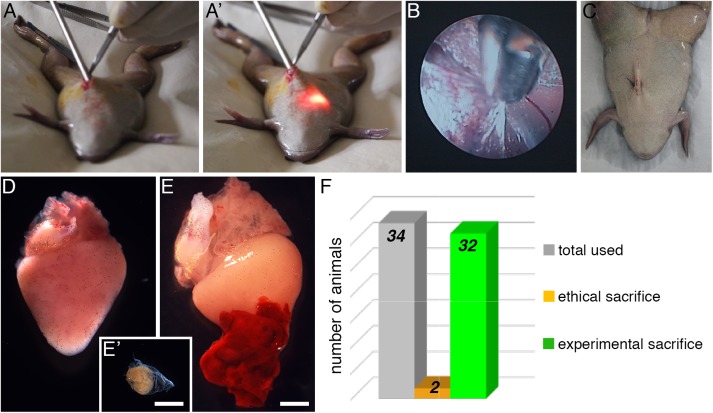
Ventricular resection endoscopic biopsy and animal survival in adult *Xenopus laevis*. (**A-C**) Key steps of the heart ventricular biopsy procedure using an endoscope. After an abdominal incision, the endoscope is inserted into the pleuroperitoneal cavity (**A**), using a xenon light source and light cable (**A’**) to search for the heart and observe the procedure internally. Biopsy forceps are used to break the falciform ligament, open the pericardial sac (**B**), then collect a calibrated piece of cardiac tissue from the apical region of the ventricle (see also S1 File). The surgery is ended (**C**) by performing a single suture to close the incision. (**D-E**) Comparison of a control non-operated heart (**D**) with an operated heart, displaying a large blood clot 1 day post-amputation (**E**), the size of biopsy tissue (**E’**) collected corresponds to approximately 4% of the ventricle. (**F**) Graphical representation of survival after the procedure, detailing total number of animals used for the full amputation procedure (n = 34), and animals ethically sacrificed (n = 2) or experimentally sacrificed at the end of the planned protocol (n = 32). *Scale bars*, 2 mm (D, E and E’).

To overcome the problems of invasive methods, we developed an endoscopy-guided protocol for resecting part of the ventricle in the adult anuran *Xenopus laevis* (Fig 1, S1 Fig and S1 File). The minimal invasive surgery itself (from incision to skin suture) took on average 5 minutes (Fig 1A–1C). Compared to a non-operated heart (Fig 1D), a blood clot quickly formed at the site of amputation (Fig 1E), demonstrating that the procedure breached the myocardial wall to reach the cavity of the ventricle. The cardiac biopsies were collected (Fig 1E’) and we estimated that approximately 4% of the ventricle volume was thus amputated.

All animals (100%) recovered from the procedure uneventfully, regardless of whether they followed the complete procedure (AMP) (Fig 1), were anesthetised but not operated (CTRL) or the surgery was stopped after opening the pericardium (SHAM).

We performed 34 endoscopic amputation procedures (S2 Table). Of these 34 animals, 2 were ethically sacrificed (at 3 and 20 hours post-surgery) due to ill health, and 32 animals (>94%) were clinically healthy until a predetermined sacrifice time (Fig 1F and S2 Table), showing no distinguishable characteristics (e.g. mobility, appetite, skin pigmentation and escape reaction) from CTRL animals. We next investigated the cardiac regenerative capacity in the adult frog heart.

### Persistent fibrosis is observed in injured frog ventricles

**Fig 2:**
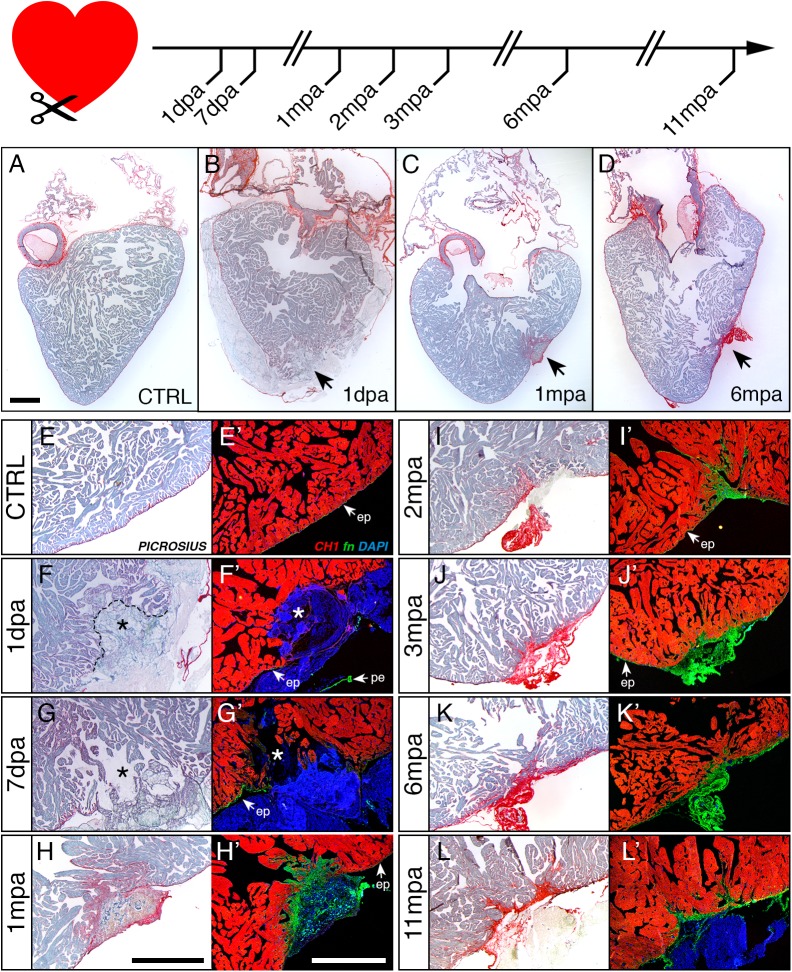
Long-term monitoring reveals persisting fibrotic scar in adult frog hearts after cardiac amputation. Top: Schematic timing of heart collection following endoscopic cardiac biopsy in adult frogs from 1 dpa up to 11 mpa. (A-D) Histology of adult heart after picrosirius (PSR) staining to label cytoplasm (i.e. cardiomyocytes) in blue and fibrous matrix (i.e. pericardium, epicardium and endocardium as well as fibrotic scar tissue) in red, for a control non-operated heart (A, CTRL), compared to heart sections at one day (B, 1 dpa), one month (C, 1 mpa) and six months (D, 6 mpa) post amputation. Black arrows indicate amputation sites; faint blue staining formed a clot around the ventricle at 1 dpa; note the presence of a low to intense red-stained scar at the site of amputation for 1 mpa and 6 mpa respectively. (EE’-LL’) Magnifications of sections stained with PSR and adjacent sections immuno-labelled for tropomyosin (CH1, red), fibronectin (fn, green) and counterstained with DAPI (nuclei, blue), for control (E-E’), and operated hearts at 1 dpa (F-F’), 7 dpa (G-G’), 1 mpa (H-H’), 2 mpa (I-I’), 3 mpa (J-J’), 6 mpa (K-K’), and 11 mpa (L-L’). PRS labelling allowed the observation of scars on each amputated section, the red labelling displaying an increase of intensity with time (E to L): a low and diffuse red staining was detectable at 1 dpa and 7 dpa around the site of amputation (*) and intensifies from 1 mpa to 11 mpa. Likewise, fibronectin immunolabelling shares a similar pattern as PSR with an increasing accumulation at the site of amputation from 1 mpa to 11 mpa (E’ to L’). White arrows: pe, pericardium; ep, epicardium. Animals: CTRL, n = 2; 1dpa, n = 1; 7dpa, n = 2; 1mpa, n = 3; 2mpa, n = 3; 3mpa, n = 2; 6mpa, n = 2, 11mpa, n = 2. Scale bars, 1 mm (A–D, E-L and E’-L’).

Cardiac histology was monitored up to 11 months post-amputation (mpa). Animals were sacrificed and hearts collected at 1 and 7 days post-amputation (dpa; Fig 2, top) and at 1, 2, 3, 6, and 11 mpa (Fig 2, top), and analysed using picrosirius (Fig 2A–2L) or immunohistochemical fibronectin and tropomyosin (Fig 2E’–2L’) labelling in order to assess both the immediate and long-term effect of injury on the ventricle. We observed that our method targeted a precise area, just above the apex, in a reproducible manner (Fig 2B–2D, arrowheads). The reproducibility of the amputation area is due to the approach angle of the endoscope to access the heart and perform the biopsy, which is comparable for each animal. Compared to a CTRL ventricle (Fig 2A, 2E and 2E’), the injured zone was clearly visible at 1 dpa (Fig 2B, 2F and 2F’) appearing as an area devoid of cardiomyocytes. In this area we observed a dense cluster of nuclei as revealed by DAPI staining (Fig 2F’) as well as a loose network of fibrous structures appearing light blue in picrosirius (Fig 2F, around the asterisk), which extended outwards (blueish haze surrounding the ventricle in Fig 2B) and corresponded to the blood clot (Fig 1E). The clot was still closing the wound at 7 dpa, when fibronectin expression started to increase in the epicardium (Fig 2G and 2G’), then it was progressively replaced by fibronectin rich scar tissue starting at 1 mpa (Fig 2C, 2H and 2H’). In the scar closing the wound, the DAPI staining progressively disappeared while the fibronectin labelling intensified through 2 mpa to 6 mpa (Fig 2I–2K and 2I’–2K’) and persisted at 11 mpa (Fig 2D, 2J–2L and 2J’–2L’). At each time-point observed, fibrosis persistence and non-continuity of the myocardial wall at the injury site demonstrated lack of regeneration.

We thus showed that the heart of the adult anuran *Xenopus laevis* is unable to regenerate, even after 11 months post-amputation (Fig 2), despite the fact that only 4% of the ventricle volume was removed (Fig 1E’).

### Cardiomyocytes are hypertrophic at the site of amputation

**Fig 3:**
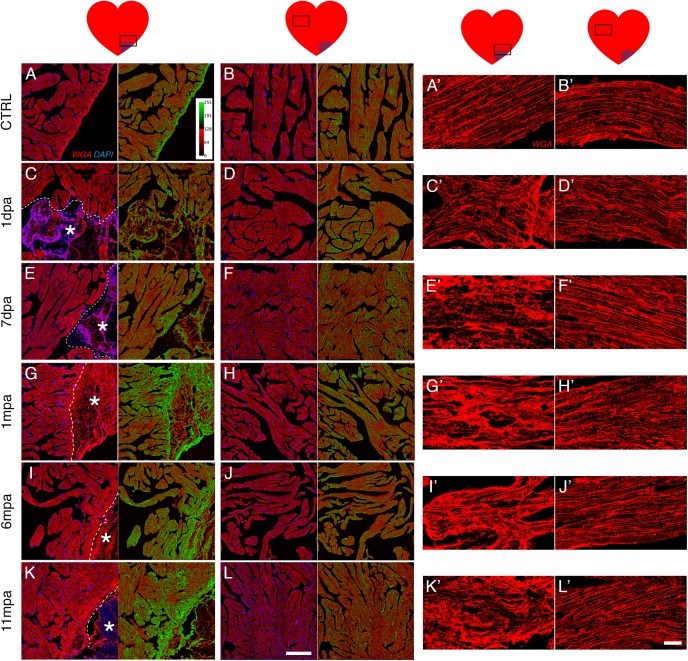
Cardiomyocytes show evidence of cell hypertrophy at the amputation site. Cardiomyocytes were observed at the site of amputation (*; see A, C, E, G, I, K and their respective magnifications A’, C’, E’, G’, I’, K’) and in a remote zone of the amputated ventricle (see B, D, F, H, J, L and their respective magnifications B’, D’, F’, H’, J’, L’), using WGA labelling as an indicator of hypertrophy. (A-L) Sections were labelled with WGA (cell membranes, red) and DAPI (nuclei, blue) for different times after amputation (1 & 7 dpa and 1, 6 & 11 mpa) and compared with a control non-amputated heart (CTRL). On the right of each picture, a post treatment of the red/WGA images allows better visualisation of the signal intensity. Note that in the control heart the myocardium showed a comparable level of labelling in the apex or in the remote zone of the ventricle (A, B), with a stronger signal in the epicardium (A). An increase of WGA labelling in the myocardium was evident from 1 mpa to 11 mpa in the vicinity of the amputation site (compare the green colour for G, I, K with C, E), whereas no differences were seen in the remote zone with the CTRL (compare the green colour for D, F, H, J, L with B). (A’-L’) Magnification of a longitudinal view of WGA-labelled cardiomyocytes: control heart showed a thin and regular WGA labelling (A’ and B’) whereas for the amputated heart, an increase of the thickness and the irregularity of the WGA signal was observed from 1 dpa up to 11 mpa compared to the control (A’) or with the WGA signal in the remote zone (see B’, D’, F’, H’, J’, L’). Animals: CTRL, n = 2; 1dpa, n = 1; 7dpa, n = 2; 1mpa, n = 3; 6mpa, n = 2, 11mpa, n = 2. Scale bars, 200 μm (A–L), 20 μm (A’–L’).

Cardiac remodelling through hypertrophy is an adaptive response to cardiac stress, ultimately leading to heart failure [2,3]. Since we observed that adult *Xenopus* heart displayed no sign of regeneration after cardiac injury, we looked for signs of hypertrophy by WGA staining (Fig 3, S2 Fig and S3 Fig). Using a filter, we transformed the images to enhance visualisation (Fig 3A–3L, right panels). Compared to CTRL hearts, there was no obvious increase in WGA staining in the vicinity of the injury site during the first week post-amputation (Fig 3C and 3E, S2 Fig). However, by 1 mpa, the injured zone displayed a clear increase in density of WGA staining (Fig 3G, S2 Fig), which persisted at 3, 6 and 11 mpa (Fig 3I and 3K, S2 Fig), meaning that more cell membrane was present in this region, and might reflect signs of cell hypertrophy with possibly associated atrophy. Increased WGA labelling was restricted to the vicinity of the amputation site since it was not observed in remote areas (compare Fig 3G, 3I, and 3K to Fig 3H, 3J and 3L, respectively). At higher magnifications the alteration of WGA staining was even more evident in longitudinal (Fig 3A'–3L’) or coronal (S3 Fig) views. Remarkably, WGA staining revealed that cardiomyocyte shape and tissue organisation were altered close to the amputation site at 1 dpa and remained altered up to 11 mpa (Fig 3C’, 3E’, 3G’, 3I’ and 3K’ and S3 Fig, top panels) compared to the control (Fig 3A’ and 3B’) and the corresponding remote areas (Fig 3D’, 3F’, 3H’, 3J’ and 3L’ and S3 Fig, bottom panels).

**Fig 4:**
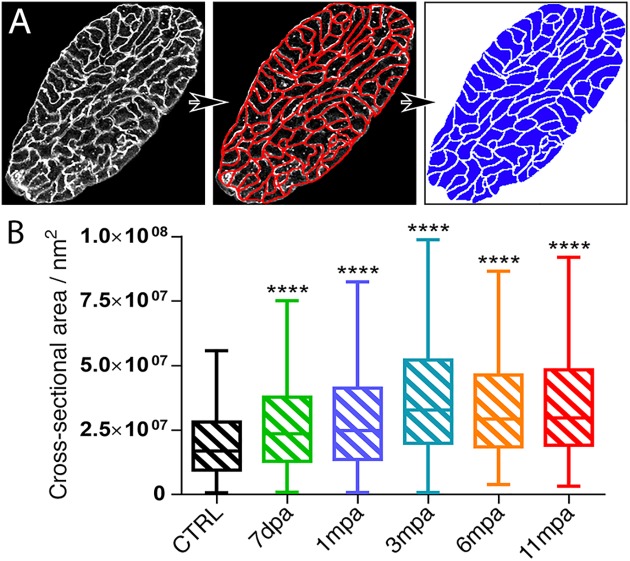
Increased cardiomyocyte size persists eleven months post amputation. (**A**) Using the WGA labelled heart pictures, the cell-membrane labelled boundaries of orthogonal view cardiomyocytes (left panel) was delineated in red (middle panel) and filled with blue colour (right panel) for non-amputated control hearts (CTRL) and different time-points post-amputation (7 dpa, 1, 3, 6 and 11 mpa). (**B**) The cross-sectional area of each cell was automatically calculated using ImageJ software. Quantification was performed on 2 or 3 independent heart sections, corresponding to minima of 300 cell areas counted for each group, and samples were compared to the CTRL. Animals: CTRL, n = 1; 7dpa, n = 1; 1mpa, n = 1; 3mpa, n = 1; 6mpa, n = 1, 11mpa, n = 1. An unpaired non-parametric t-test (Mann Whitney) was performed: ****, p<0.0001.

**Fig 5:**
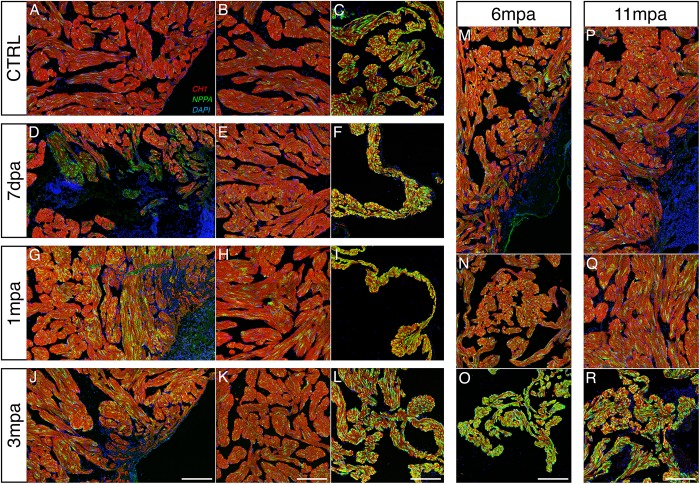
Long lasting cardiac hypertrophy occurs in amputated frog heart. Cardiomyocytes were observed at the site of amputation (**A, D, G, J, M, P**) and in a remote zone of the amputated ventricle (**B, E, H, K, N, Q**), using immuno-detection of natriuretic peptide A as an indicator of cardiac hypertrophy. Sections were labelled for tropomyosin (CH1, red), natriuretic peptide A (NPPA, green) and DAPI-counterstained nuclei (blue) at different times-points after amputation (7 dpa and 1, 3, 6 & 11 mpa) and compared with a control non-amputated heart (CTRL). In the vicinity of the amputation site, a weak NPPA signal was detected in CTRL (**A**), slightly increased at 7 dpa (**D**), whereas a clear increase of NPPA labelling in the myocardium was evident from 1 mpa to 11 mpa (**G, J, M, P**). In the remote zone, the NPPA labelling seemed appeared to slightly increase over time compared to the CTRL (compare **B, E, H, K, N, Q**). As a labelling control, all samples displayed an intense NPPA signal in atrium (**C, F, I, L, O, R**), since *nppa* is highly expressed in this tissue. Animals, CTRL, n = 1; 7dpa, n = 1; 1mpa, n = 1; 3mpa, n = 1; 6mpa, n = 1, 11mpa, n = 1. *Scale bars*, 200 μm (A–R).

Thus, in order to determine the presence of cell hypertrophy, individual cell areas of orthogonal view cardiomyocytes were quantified near the amputation site (Fig 4). We observed that at all time-points post-amputation monitored, the cross-sectional area of cardiomyocytes was enlarged compared to the control, concluding a hypertrophic state of the cardiomyocytes in the vicinity of the amputated zone. Further, to confirm these observations, we performed an immuno-staining against natriuretic peptide A (NPPA), another known marker of hypertrophy, on heart sections at the same time-points post-amputation described above (Fig 5). From 1 mpa, we observed a clear increase of NPPA signal in the vicinity of the amputation site that remained present at 11 mpa, whereas signal was weak for the CTRL heart. In the remote zone, NPPA staining appeared to slightly increase in amputated hearts, suggesting an impact on the whole heart following injury.

Together these data revealed an induction of persistent cardiomyocyte hypertrophy after heart injury in adult *Xenopus*. Moreover, cellular and tissue disorganisation occurred concomitantly with the hypertrophy.

### Striated cardiomyocyte disorganisation is observed at the amputation site

**Fig 6:**
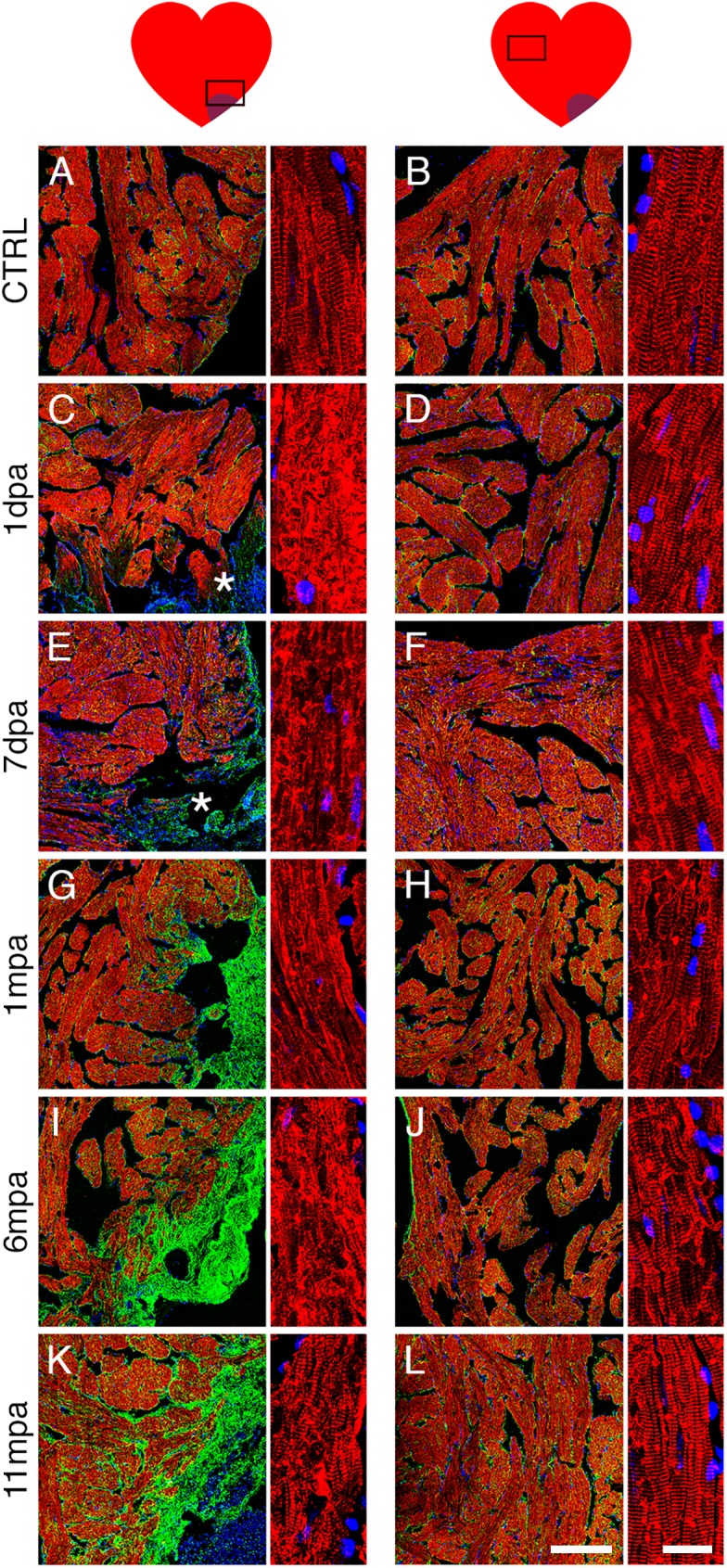
Sarcomeric organisation of cardiomyocytes is deteriorated near the amputation site. (**A-L**) Immuno-labelled sections for tropomyosin (CH1, red), fibronectin (fn, green) and DAPI-counterstained nuclei (blue), for a control non-amputated heart (CTRL) compared to 1 & 7 dpa, and 1, 6, and 11 mpa). Sarcomere organisation is observed at the amputation site (**A**, **C**, **E**, **G**, **I**, **K**, and their respective magnification) and in a remote zone of the amputated ventricle (**B**, **D**, **F**, **H**, **J**, **L**, and their respective magnification). The tropomyosin signal revealed a thin and well-organised striated structure of the cardiomyocytes for the CTRL heart. In contrast, at the site of amputation, the sarcomere organisation was completely disorganised at 1 dpa, lost at 7 dpa, partially recovered but not completely at 6 mpa and 11 mpa respectively (compare left magnifications). No evident change was seen between CTRL and AMP hearts in the remote zone (compare right magnifications). Note the general increase of the fibronectin staining around the cardiomyocytes for 1 mpa, 6 mpa and 11 mpa both at the site of amputation and in the remote zone. An asterix (*) marks the amputation site for time-points where fibrotic scar is not obvious. Animals: CTRL, n = 2; 1dpa, n = 1; 7dpa, n = 2; 1mpa, n = 3; 6mpa, n = 2, 11mpa, n = 2. *Scale bars*, 200 μm (**A–L**), 20 μm (magnification).

To further investigate cardiomyocyte structure and organisation, we observed the dynamics of tropomyosin labelling at higher magnification near the site of amputation, where fibrosis was observed, and in remote zones (Fig 6). Tropomyosin is one of the central elements of the sarcomeric unit, regulating interactions between actin and myosin in the muscle sarcomere. These sarcomeric units organise as a series of bands visible along the muscle fibres, and are responsible for the striated appearance of the cardiomyocytes. A regular striated cardiomyocyte structure was present throughout CTRL hearts (Fig 6A and 6B, right panels). However, at 1 dpa near the amputation site, a marked increase of tropomyosin labelling was observed in some fibres (Fig 6C, left panel) and a considerable disorganisation of sarcomere structure was evident (Fig 6C, right panel). At 7 dpa the striated structure was absent (Fig 6E, right panel). Sarcomere structure was then partially restored at 1 and 6 mpa (Fig 6G–6I, right panels), but did not completely recover to a state as orderly as CTRL at 11 mpa (Fig 6 compare A with K, right panels). Conversely, at each time-point post-amputation, the striated structure in the remote zone was similar to CTRL, as no sign of disorganisation was observed (Fig 6B, 6D, 6F, 6H and 6J). Note, the myocardium in the remote zone appeared to contain more interstitial fibronectin at 1, 6 and 11 mpa, highlighted by a “yellowish” colour all around the cardiomyocytes (Fig 6 compare B, D, F to H, J and L). As supplemental controls, we performed fibronectin, WGA and NPPA immuno-labelling (S4A-S4N Fig) on SHAM hearts collected 11 months post-operation (SHAM 11 mpa), and its corresponding CTRL (CTRL 11 mpa). We showed that there was no difference in all immuno-labelling performed, between CTRL 11 mpa, SHAM 11 mpa (S4A–S4N Fig) and the 11-months “younger” CTRL (i.e. the 11 months-less non-amputated control heart) used in other figures (compare S4A–S4N Fig to Fig 3A and 3B, Fig 5A–5C, and Fig 6A and 6B). Interestingly, cross-sectional cardiomyocyte areas for the CTRL 11 mpa were increased compared to the “younger” CTRL (S4O Fig), revealing a probable cell hypertrophy induced by aging. Nevertheless, compared to CTRL 11 mpa and SHAM 11 mpa that had no difference in cross-sectional cardiomyocyte area, a significantly increased cell area in AMP 11 mpa strongly indicated injury-induced hypertrophy (S4O Fig).

Together with cardiomyocyte hypertrophy (Figs 3, 4 and 5), altered striated structure (Fig 6) suggested that myocardium was largely disorganised locally, at the amputation site. Furthermore, this disorganisation was persistent and the sarcomeric striated structure was not fully recovered at 11 mpa.

### Markers of fibrosis, hypertrophy and inflammation are up regulated during the first week post-amputation

**Fig 7:**
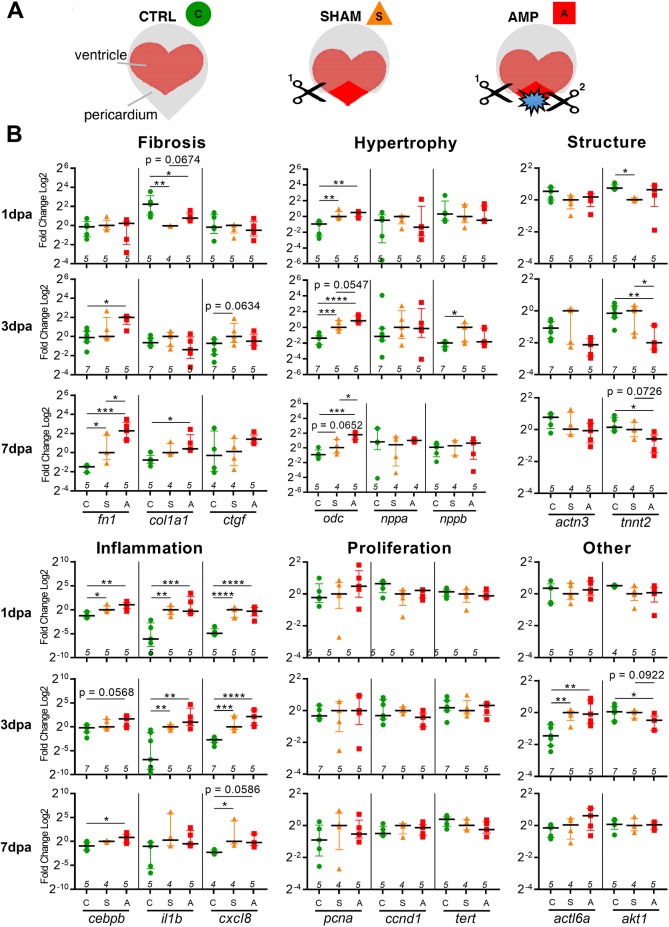
Short-term transcriptional responses in adult frog heart after cardiac injury display evidence of a scarring process. (**A**) Heart ventricles from different experimental conditions were collected for RT-qPCR analysis: control non-operated hearts (CTRL), SHAM-operated hearts where only the pericardium was opened (scissor 1, SHAM), and amputated hearts where a ventricle biopsy was performed following pericardium opening (scissor 1 & 2, AMP). (**B**) RNA extraction was performed on whole heart ventricles, collected at days 1, 3 and 7 post-amputation followed by RT qPCR to quantify gene expression. Gene markers of fibrosis (*fn1*, *col1a1*, *ctgf*), hypertrophy (*odc*, *nppa*, *nppb*), cellular structure (*actn3*, *tnnt2*), proliferation (*pcna*, *ccnd1*, *tert*), inflammation (*cebpb*, *il1b*, *cxcl8*), *actl6a* and *akt1* were monitored. Aligned dot plot with median and interquartile range, n≥4 for each group with exact n displayed above the x-axis. Normalisation was performed using the geometric mean of two reference genes (*smarcd1*/*smn2*), fold-change is shown in log2 scale, respective to SHAM for each gene at each individual time-point. *, p < 0.05, **, p < 0.01, ***, p < 0.001, ****, p < 0.0001.

We next investigated the immediate molecular responses of hearts from CTRL, SHAM and AMP groups (Fig 7A) during the first week following amputation (Fig 7B). We monitored genes known to be involved in the cardiac scarring process, such as fibrosis (*fn1*, *fibronectin1*; *col1a1*, *collagen type 1 alpha 1*; *ctgf*, *connective tissue growth factor*), hypertrophy (*odc*, *ornithine decarboxylase; nppa*, *natriuretic peptide A; nppb natriuretic peptide B*), and inflammation (*cebpb*, *CCAAT/enhancer binding protein beta*; *il1b*, *interleukin 1b*, *cxcl8*, *interleukin 8*).

In AMP animals, *fn1* expression significantly increased by 3 dpa compared to CTRL, and remained higher than both CTRL and SHAM animals at 7 dpa. A significant increase was also observed for *col1a1* expression compared to CTRL at 7 dpa, whereas change in *ctgf* expression was not significant. The hypertrophic marker *odc* was significantly up regulated at each time observed for AMP compared to CTRL, and compared to SHAM at 7 dpa as well as for SHAM compared to CTRL at 1 and 3 dpa. In addition to *odc*, the expression of hypertrophic markers *nppa* and *nppb* was also analysed. It is known that nppa is highly expressed in the atria, however we were able to analyse any change in expression caused by cardiac injury in the ventricle alone, since our samples have had the atria removed. Unfortunately, in the short time-frame observed, the expression of *nppa* and *nppa* remained unchanged and were not upregulated in the first week following amputation. In accordance with this observation, immuno-labelling for NPPA was clearly increased from 1 mpa in the ventricle near the amputation site (Fig 5). Taken together, our data showed that expression markers of fibrosis and hypertrophy were increased early in amputated hearts and fitted with the long-term observation of fibronectin deposition and cardiac hypertrophy in injured hearts.

Similar increased expression of inflammatory markers was observed for AMP and SHAM conditions compared to CTRL. Therefore opening the pericardium in SHAM animals and ventricle resection in AMP animals provokes a similar inflammatory response, at least concerning the genes that we monitored.

### No molecular evidence of increased cell proliferation or cardiomyocyte differentiation during the first week post-amputation

Cardiomyocyte proliferation is used as an index of the regenerative potential of the heart following cardiac injury [7,8]. We thus quantified expression levels of cell proliferation markers, such as the mitotic factor *pcna* (*proliferating cell nuclear antigen*), the cell cycle marker *ccnd1* (*cyclin D1*), and *tert* (*telomerase reverse transcriptase*) (Fig 7B). For all genes tested, no significant change of expression was observed between CTRL, SHAM or AMP situations at any time-point monitored.

Two factors known to be involved in differentiation processes were also investigated: *actl6a* (*actin like 6a*, also known as *baf53a*), a SWI/SNF BAF-like complex member thought to prevent activation of differentiation programs [39,40], and *akt1* (*v-akt murine thymoma viral oncogene homolog 1*) documented as essential for initiation of muscle differentiation [41], also shown to improve fibroblasts conversion into functional cardiomyocytes [42] (Fig 7B). At 3 dpa, *actl6a* was up regulated in SHAM and AMP compared to CTRL, whereas *akt1* was significantly down regulated in AMP compared to CTRL and lower in AMP than SHAM (p = 0.0922).

These results indirectly suggested that the adult ventricle was unable to mount a proliferative response and to differentiate novel cardiomyocytes after injury.

### Expression of sarcomere components is altered early after amputation

Given that we observed an alteration of the striated structure of cardiomyocytes at the injured site (Fig 6), we quantified the expression of genes encoding proteins of the sarcomeric apparatus *tnnt2* (*troponin T type 2*) and *actn3* (*actinin alpha 3*) (Fig 7B). Both genes encode proteins that participate in the sarcomeric unit assembly, are essential to maintain the Z-disc structure, and play a role in muscle contraction [43–45]. Interestingly, these genes were down regulated in AMP compared to CTRL at 3 and 7 dpa, especially *tnnt2*, which was also down regulated compared to SHAM at 3 dpa (Fig 7B). This result corresponded with the lasting altered sarcomeres observed in cardiomyocytes close to the amputation site (Fig 6). Alternately, this could be due to an increase in the ratio of non-myocytes (infiltrating cells or fibroblasts) to cardiomyocytes.

## Discussion

Investigating cardiac regenerative potential is of importance to develop therapy. In adult humans, there is an absence of cardiomyocyte regeneration following infarction. Instead, a fibrotic scar replaces the injured tissue, negatively impacting cardiac function and possibly leading to heart failure [2,3]. Model organisms are increasingly used and developed to understand the processes involved in regeneration, scar formation and hypertrophy in hope to reverse the adverse effects of myocardial injury in humans [4].

Currently, our knowledge of cardiac regeneration and scarring processes is dominated by data obtained from zebrafish and mouse models [4–9,30,31,46]. Vertebrate cardiac regenerative capacity has recently been discussed in an evolutionary and comparative context [26]. These authors highlight the potential of using alternate models to explore the consequences of heart injury. Remarkably, with the exception of medaka that has not regenerated the ventricle by 60 dpa [47], it is between the evolutionary separated teleost (zebrafish) and mammal (mouse) that adult capacity to regenerate the heart is lost, but the mechanisms underlying the proximal (i.e. physiological) and ultimate (i.e. evolutionary) [48] causes of this difference remain unknown. This is why amphibians, positioned between these two models in the vertebrate evolutionary tree, are of particular interest. Even though novel data has been obtained for urodeles [49–51], knowledge remains scarce for anurans. To update and develop the limited data from the initial 1870s and 1960s studies [21–23], we introduced *Xenopus laevis* as a contemporary model to analyse cardiac injury, using a method designed to be less invasive than those already described.

Presently, the main procedures adopted to cause cardiac injury in vertebrate models are cryoinjury or tissue removal by resection that damage between 10% and 30% of the ventricular chamber, and left anterior descending (LAD) coronary artery ligation that causes approximately 60% to 75% of cardiomyocyte loss. Interestingly, LAD is the more popular method for investigating cardiac repair in non-regenerating warm-blooded species. Whereas in the regenerating cold-blooded species, physical cardiac damage by heat, cold and tissue removal methods are mainly used [26].

When assessing available heart injury methods applicable to amphibians, important factors to consider are the fact that damage to the ventral abdominal vein can be detrimental [33] and that a pectoral girdle and cartilaginous sternum cover the heart preventing easy access [52]. In urodele studies, an open surgery method was used to perform a ventricular amputation [16,17,50]. However, it was shown that the procedure used to gain access to the ventricle was sufficient to induce fibrosis and cardiomyocyte cell cycle re-entry in 1 day-old mice [31]. It is therefore possible that open surgery has a similar impact on amphibian heart. Further, a general problem associated with any method is the invasiveness of the surgery, which can cause significant stress during and after the operation. As it has been shown that stress can be detrimental to heart regeneration in zebrafish [53], this might be a source of artefactual responses during the regenerative process.

In an endeavour to reduce stress, we optimised a minimally invasive endoscopy-guided surgical procedure and applied it to investigate the response to injury after resection of adult *Xenopus* heart ventricle. Compared to the methods described above, using endoscopy to cause cardiac injury can be considered less severe, with minimal trauma, discomfort and stress [33,54]. As previously demonstrated, this method is rapid, allows live visualisation of all coelomic organs, including their size and colour, providing spatial awareness around structures from a 3-mm single paramedian entry point [33,54]. In addition, the amount of tissue collected is standardised by biopsy forceps size, increasing the reproducibility of the extent of inflicted damage; we estimated that 4% of ventricle volume was amputated in our experiments. Standardisation has the benefit of eliminating potential variation in the results arising from technical difficulties like different injury sizes, an issue that was proposed to explain different cardiac regeneration results observed in mice [9,30,31,46]. If amphibian endoscopy is performed correctly, complications are rare, although iatrogenic trauma can occur, as can buoyancy issues if CO_2_ remains from insufflation after suture [33]. We did not experience any endoscopy related difficulties during these experiments; 100% of animals survived the optimised procedure itself and thereafter >94% survived until the predetermined sacrifice time-point.

Applying our technique, we showed that adult *Xenopus* heart mounts a similar inflammatory response in SHAM and in AMP conditions when compared to CTRL (Fig 7B). Given the important roles of the pericardium in cardiac function [55] it is probably not surprising that frog ventricles display marked signs of inflammation, even in the SHAM group.

Another feature of the early response of *Xenopus* ventricle to injury was the up regulation of genes participating in fibrosis (*fn1*, *col1a1*) and hypertrophy, (*odc*) (Fig 7), two main signs of a scarring process [5,56]. At the tissue level, fibrosis and hypertrophy were evident and long lasting. Fibrotic deposition and hypertrophy increased over time, becoming progressively more invasive at the amputation site, notably from 1 mpa (Figs 2, 3, 4, 5 and 6, and S2 and S3 Figs). Further, from 1 mpa onwards, interstitial fibrosis also increased in remote areas of the heart, in cardiac tissue not directly affected by the injury (Fig 6). Conversely, hypertrophy remained in the vicinity of the injury (Figs 3, 4 and 5, and S2 and S3 Figs). Importantly, myocardial wall continuity was not restored even 11 months after amputation, with fibronectin scar still filling gaps in the tissue (Fig 1L and 1L’, Fig 6K, left panel).

Concurrent with fibrosis and hypertrophy, we observed marked sarcomere disorganisation around the amputation site (Fig 6). This disassembly took place as early as 1 dpa (Fig 6), and was associated with a significant decrease in expression of genes coding for two important actors of the sarcomere, *actn3* and *tnnt2* (Fig 7). Although data is limited regarding cardiac muscle, *actn3* plays a role in skeletal muscle sarcomere structure, organisation and function. In mammals, reduced ACTN3 has been shown to decrease muscle mass, lower contractile properties and increase susceptibility to muscle damage [43,57,58]. In humans and zebrafish, TNNT2 function is known to be required for proper cardiac sarcomere assembly and heart contractility [59–62]. Therefore, decreased *actn3* and *tnnt2* expression after injury in *Xenopus* heart is likely involved in the observed sarcomere disassembly and might result in decreased contractility in the vicinity of the injured area.

Sarcomeric disorganisation preceding the proliferative step is an initial feature of cardiac regeneration in zebrafish and neonatal mice, occurring in the first week following injury [7,8]. Similarly, initial disassembly of the sarcomere structure was also observed, preceding mono-nucleated cardiomyocyte division in adult mice heart stimulated to regenerate [63]. However, in *Xenopus*, we did not observe a concomitant change in the expression levels of genes encoding for the proliferative factors *pcna*, *ccnd1* and *tert* (Fig 7). Furthermore, if partially recovered at 1 mpa, sarcomeric disarray was not transient as in zebrafish, still being present at 6 and 11 mpa (Fig 6). Our data thus suggested that sarcomeric disorganisation in injured adult *Xenopus* heart did not promote cell renewal but rather reflected a return to a foetal-like phenotype as has been reported to occur in failing adult mammalian hearts [64].

We also observed that *akt1* expression was significantly decreased 3 dpa (Fig 7). This gene encodes for an integrator of cell signalling, with pleiotropic actions. In mammalian cardiomyocytes, AKT activity is literally involved in all aspects of cell function including contractility, survival, metabolism, growth and hypertrophy; at the organ level it has cardio-protective effect against pressure-overload induced heart failure [65]. This factor was also shown to improve fibroblast conversion into cardiomyocyte *in vitro* [42]. Given the central role of AKT activity in heart tissue, it is thus not inconceivable that decreased *akt1* expression (Fig 7) might adversely affect the regenerative process.

These observations demonstrated that no regeneration took place up to almost one-year post-amputation in the frog heart. Hence, *Xenopus* differs from urodele amphibians and zebrafish that do regenerate their heart after damage.

Multiple authors have proposed that during evolution, the capacity to regenerate the heart was lost concurrently with the gain of homeothermy, and that features linked with poikilothermy such as low metabolic rate, low cardiac workload and trabecular organisation of the heart, are permissive for regeneration [26,66–68]. Our results show that the trabecular heart of the cold-blooded *Xenopus laevis* cannot regenerate and therefore are at odds with this hypothesis. In fact, the way that *Xenopus* heart responds to damage is reminiscent to that of warm-blooded adult mammals: no regeneration, hypertrophy to compensate the lost contractile ability, persistent scarring to maintain organ continuity and prevent rupture of the ventricular wall [28]. This raises an interesting question: was cardiac regenerative capacity lost in basal tetrapods and regained in urodeles or lost independently in anurans (or at least *Xenopus laevis*) and amniotes? The fact that, among teleosts, the medaka is also known to be unable to regenerate its heart ventricle might favour the later [47]. Comparative studies will be needed to clarify this point.

The long-term disarray of sarcomeric structure in adult *Xenopus* heart, associated with the absence of regenerative capacity and persistent fibrosis and cardiomyocyte hypertrophy, suggested an alteration of the structural and functional properties of the myocardial tissue. It would be interesting to monitor heart function in frogs before, immediately after, and at different time points following amputation. This might be investigated using advanced echocardiography with modern speckle-tracking analyses to detect changes in cardiac performance, since it has successfully been applied to zebrafish to monitor cardiac functional recovery after amputation [69]. Other techniques can be applied like positron emission tomography (PET) as tested on pigs following induced myocardial infarction [70] or optical coherence tomography, which was used on tadpoles [71].

## Conclusion

**Fig 8:**
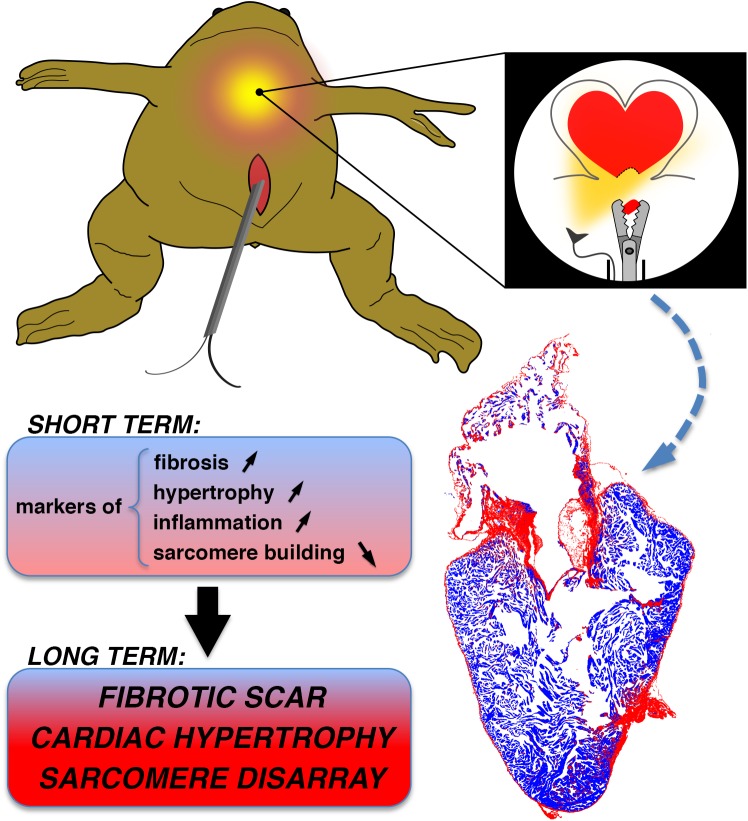
Endoscopic ventricle biopsy procedure in adult frogs induces a fibrous scar with absence of heart regeneration. Endoscopic procedure in anesthetised frog allows “in situ” live visualisation of the operating field and collection ventricle tissue in the apical region using biopsy forceps. The resulting outcome for the frog heart is the induction of scar related gene expression during the first week post-amputation (SHORT TERM) and the establishment of a persistent fibrous scar, cardiac hypertrophy and sarcomere disorganisation at the amputation site, without the capacity for the cardiac tissue to regenerate, even after almost one year (LONG TERM).

In addition to a novel cardiac amputation procedure, we propose a contemporary vertebrate model with absence of heart regeneration to study myocardial injury and repair after tissue removal (Fig 8). This should provide a valuable future tool to explore the cellular and molecular mechanisms that underlie the scarring process after heart injury. Data obtained in our *Xenopus* model of cardiomyocyte hypertrophy could also prove relevant for translational medicine including hypertrophy regression. Moreover, the endoscopy technique, potentially transferrable to other species in the future, could help in translating the collected information into therapies aimed at salvaging and repairing failing human hearts.

## Supporting information

S1 Fig Video recording screen shots of endoscopy-guided heart resection in *Xenopus*. (**A**) A surgically prepared *Xenopus* in dorsal recumbence on the operating table with endoscope inserted inside. (**B**) The falciform ligament (light gold pattern) covering the heart (silver pattern). (**C**) The falciform ligament was broken (black arrow) to reveal the pericardium-covered heart (white arrow). (**D**) The pericardium was opened. (**E**) The heart was grabbed with the biopsy forceps. (**F**) The site of heart amputation (white arrow). (See S1 File).
(TIF)

S2 Fig Increasing cell hypertrophy occurs at the amputated site of adult frog heart. Sections labelled to reveal membranes (WGA, red), cardiomyocytes (CH1, green) and nuclei (DAPI, blue), for a control non-amputated heart (CTRL) compared to 1 and 7 dpa, and 1, 2, 3, 6, and 11 mpa. A progressive extension and intensification of WGA labelling, which is an indication of hypertrophy, was observed in cardiomyocytes localised around the amputation site: At 1 dpa and 7 dpa, hypertrophy was not detectable in the vicinity of amputation site (*), whereas an increase of WGA labelling was observed starting at 1 mpa and enlarging and intensifying up to 11mpa. Animals: CTRL, n = 2; 1dpa, n = 1; 7dpa, n = 2; 1mpa, n = 3; 2mpa, n = 3; 3mpa, n = 2; 6mpa, n = 2, 11mpa, n = 2. *Scale bars*, 500 μm.
(TIF)

S3 Fig Coronal view reveals hypertrophic cardiomyocytes at the amputation site. Magnification of coronal views of WGA-labelled cardiomyocytes observed at the site of amputation (**top**) and in a remote zone of the amputated ventricle (**bottom**), for a control non-amputated heart (CTRL) compared to different times after amputation (1 and 7 dpa and 1, 6 and 11 mpa). As in Fig 3, an increase of the thickness of the WGA signal was observed starting from 1 mpa and still present at 11 mpa compared to the control, but no difference was evidenced in the remote zone. Animals: CTRL, n = 2; 1dpa, n = 1; 7dpa, n = 2; 1mpa, n = 3; 6mpa, n = 2, 11mpa, n = 2. *Scale bars*, 20 μm.
(TIF)

S4 Fig No fibrosis or hypertrophy is observed in the heart of CTRL and SHAM frogs after eleven months. Cardiomyocytes were observed after 11 months for control non-amputated (CTRL 11mpa, **A-C** and **G-J**) and SHAM-operated hearts (SHAM 11mpa, **D-F** and **K-N**), near the ventricle border at the equivalent amputation site (**A-F**) and in a remote zone of the ventricle (**G-N**). Immuno-labelled sections for tropomyosin (CH1, red), fibronectin (fn, green) and DAPI-counterstained nuclei (blue), for CTRL (**A**, **G**, and corresponding magnification) and SHAM (**D**, **K**, and corresponding magnification) showed no difference in sarcomere organisation at the border or in a remote zone of the ventricle. On magnifications, the tropomyosin signal revealed a thin and well-organised striated structure of the cardiomyocytes for CTRL and SHAM hearts. Using WGA labelling (cell membranes, red) and DAPI (nuclei, blue), no difference was observed between CTRL and SHAM at the ventricle border (compare **B** and **E**) or in the remote zone (compare **H** and **L**). On the right of each picture, a post treatment of the red/WGA images allows better visualisation of the signal intensity (**B’**, **E’**, **H’** and **L’**). Note that the myocardium showed a comparable level of labelling in the border or in the remote zone of the ventricle, with a stronger signal in the epicardium. Immuno-labelled sections for tropomyosin (CH1, red), natriuretic peptide A (NPPA, green) and DAPI-counterstained nuclei (blue), confirmed the absence of hypertrophic signal in CTRL 11mpa and SHAM 11mpa hearts (compare **C** and **F** for the border, **I** and **M** for the remote zone). The NPPA signal in the atrium (**J** and **N**) was used as a labelling control, as *nppa* is highly expressed in this tissue. (**O**) The cross-sectional area of WGA-labelled cardiomyocytes was compared showing no difference between CTRL 11mpa and SHAM 11mpa, whereas cell area was significantly increased for AMP 11mpa, revealing hypertrophic cardiomyocytes at the site of amputation. A slight increase was also seen between both CTRLs separated by 11 months, which suggested an aging effect. Count was performed on 2 or 3 independent heart sections, corresponding to minima of 300 cell areas for each group. An unpaired non-parametric t-test (Mann Whitney) was performed: ****, p<0.0001; ns, non-significant. *Scale bars*, 200 μm (**A–N**), 20 μm (magnification).
(TIF)

S1 Table Primer sequences used for Real-Time q-PCR experiments. For each primer pair, the forward and reverse sequences are given. Most genes studied were known as a single copy when this study was conducted except *cebpb* and *ccnd1*; these primers allowed the amplification of both *cebpb* homeologs but only a single *ccnd1* homeolog. Target of *Xenopus laevis* long (.L) or short chromosome (.S) follows the gene name.
(DOCX)

S2 Table Detail of experimental design listing for each independent amputation series and the different time points used for sample collection. The total number of adult *Xenopus laevis* that followed the complete endoscopic procedure and whether they were used for a long-term or short-term analysis.
(TIF)

S1 File Key Steps during the endoscopy-guided cardiac amputation in *Xenopus laevis*. A view of a frog on an operating table (white arrow) with an endoscope inserted inside and the in situ live visualisation on a screen (black arrow). The next clips show the falciform ligament being opened, the pericardium opened and a piece of the heart amputated during the ventricular diastole. The wound is then closed using a single suture. (Videos are courtesy of Norin Chai, DVM, MSc, PhD, Paris, France.)
(WMV)
