# Supplementary figures and images for: Persistent fibrosis, hypertrophy and sarcomere disorganisation after endoscopy-guided heart resection in adult Xenopus
Source: PLoS One. 2017 Mar 9;12(3):e0173418. doi: 10.1371/journal.pone.0173418 (PMC5344503; doi:10.1371/journal.pone.0173418)

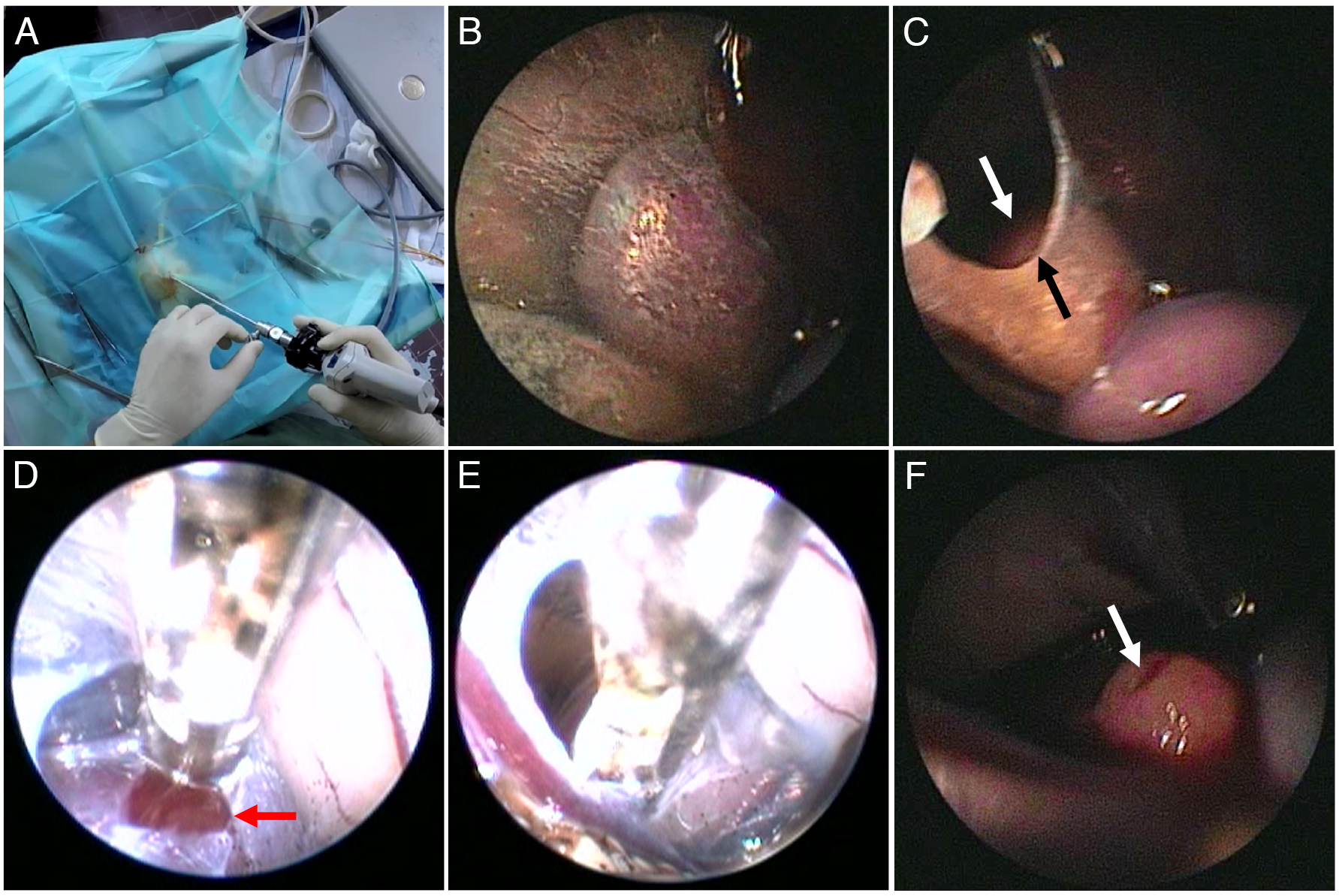

Supplement: S1 Fig — (A) A surgically prepared Xenopus in dorsal recumbence on the operating table with endoscope inserted inside. (B) The falciform ligament (light gold pattern) covering the heart (silver pattern). (C) The falciform ligament was broken (black arrow) to reveal the pericardium-covered heart (white arrow). (D) The pericardium was opened. (E) The heart was grabbed with the biopsy forceps. (F) The site of heart amputation (white arrow). (See S1 File). (TIF) [file pone.0173418.s001.tif]

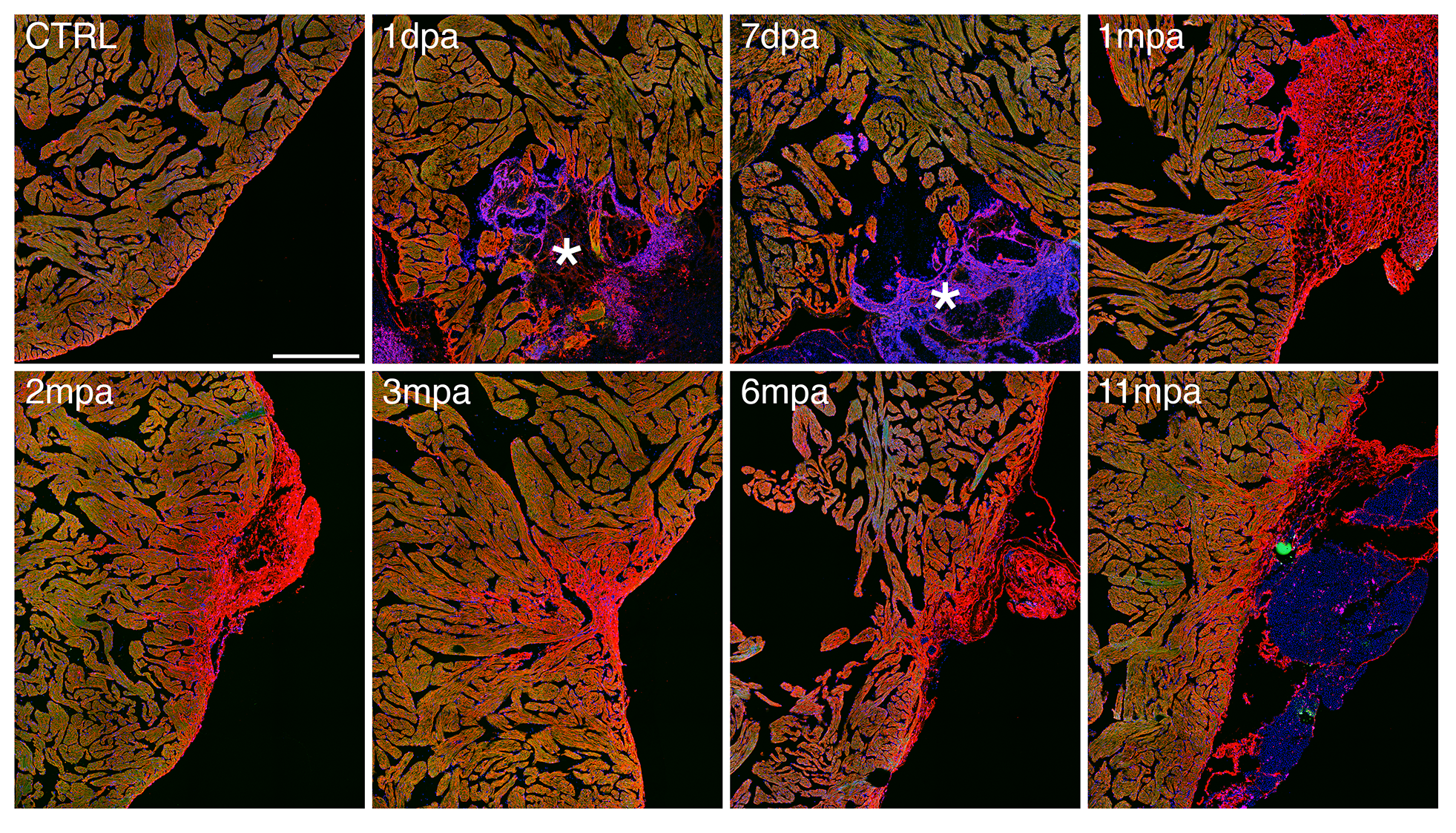

Supplement: S2 Fig — Sections labelled to reveal membranes (WGA, red), cardiomyocytes (CH1, green) and nuclei (DAPI, blue), for a control non-amputated heart (CTRL) compared to 1 and 7 dpa, and 1, 2, 3, 6, and 11 mpa. A progressive extension and intensification of WGA labelling, which is an indication of hypertrophy, was observed in cardiomyocytes localised around the amputation site: At 1 dpa and 7 dpa, hypertrophy was not detectable in the vicinity of amputation site (*), whereas an increase of WGA labelling was observed starting at 1 mpa and enlarging and intensifying up to 11mpa. Animals: CTRL, n = 2; 1dpa, n = 1; 7dpa, n = 2; 1mpa, n = 3; 2mpa, n = 3; 3mpa, n = 2; 6mpa, n = 2, 11mpa, n = 2. Scale bars, 500 μm. (TIF) [file pone.0173418.s002.tif]

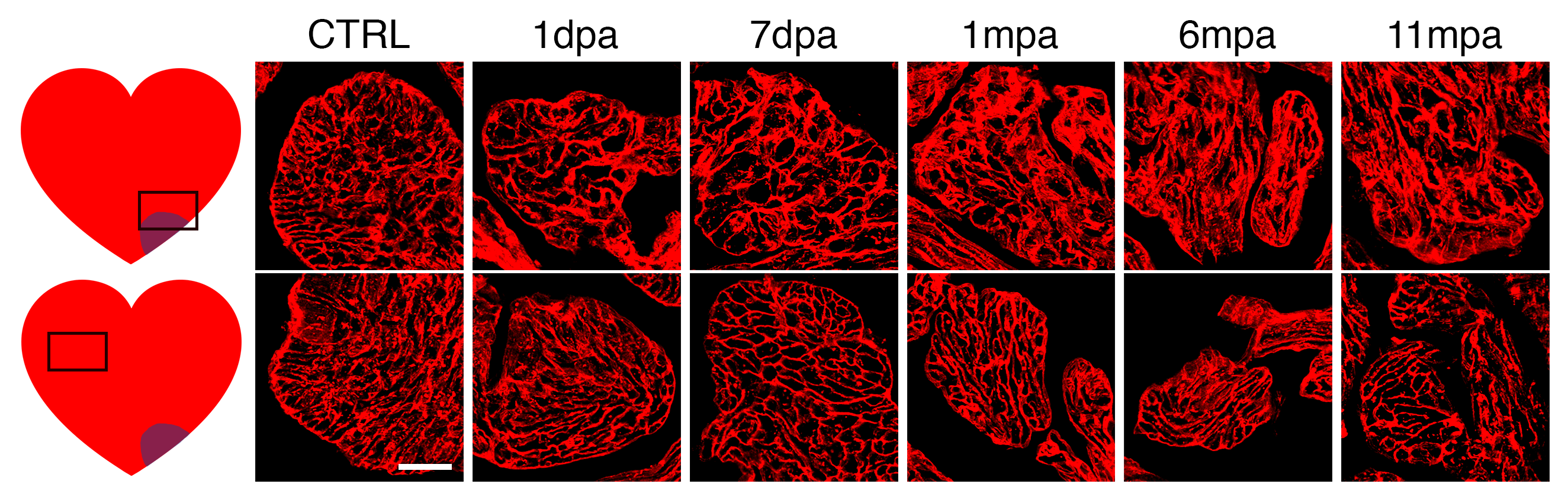

Supplement: S3 Fig — Magnification of coronal views of WGA-labelled cardiomyocytes observed at the site of amputation (top) and in a remote zone of the amputated ventricle (bottom), for a control non-amputated heart (CTRL) compared to different times after amputation (1 and 7 dpa and 1, 6 and 11 mpa). As in Fig 3, an increase of the thickness of the WGA signal was observed starting from 1 mpa and still present at 11 mpa compared to the control, but no difference was evidenced in the remote zone. Animals: CTRL, n = 2; 1dpa, n = 1; 7dpa, n = 2; 1mpa, n = 3; 6mpa, n = 2, 11mpa, n = 2. Scale bars, 20 μm. (TIF) [file pone.0173418.s003.tif]

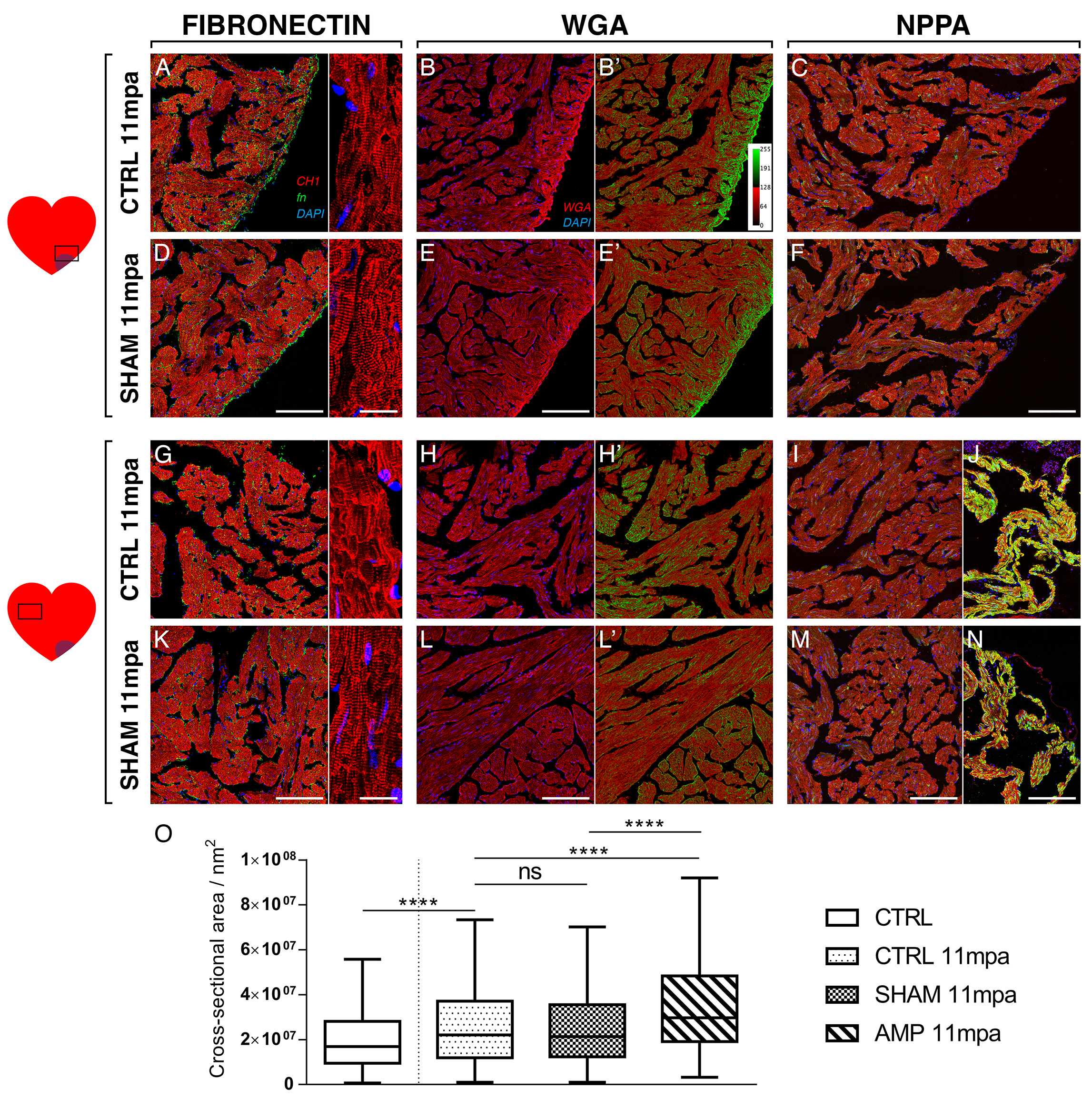

Supplement: S4 Fig — Cardiomyocytes were observed after 11 months for control non-amputated (CTRL 11mpa, A-C and G-J) and SHAM-operated hearts (SHAM 11mpa, D-F and K-N), near the ventricle border at the equivalent amputation site (A-F) and in a remote zone of the ventricle (G-N). Immuno-labelled sections for tropomyosin (CH1, red), fibronectin (fn, green) and DAPI-counterstained nuclei (blue), for CTRL (A, G, and corresponding magnification) and SHAM (D, K, and corresponding magnification) showed no difference in sarcomere organisation at the border or in a remote zone of the ventricle. On magnifications, the tropomyosin signal revealed a thin and well-organised striated structure of the cardiomyocytes for CTRL and SHAM hearts. Using WGA labelling (cell membranes, red) and DAPI (nuclei, blue), no difference was observed between CTRL and SHAM at the ventricle border (compare B and E) or in the remote zone (compare H and L). On the right of each picture, a post treatment of the red/WGA images allows better visualisation of the signal intensity (B’, E’, H’ and L’). Note that the myocardium showed a comparable level of labelling in the border or in the remote zone of the ventricle, with a stronger signal in the epicardium. Immuno-labelled sections for tropomyosin (CH1, red), natriuretic peptide A (NPPA, green) and DAPI-counterstained nuclei (blue), confirmed the absence of hypertrophic signal in CTRL 11mpa and SHAM 11mpa hearts (compare C and F for the border, I and M for the remote zone). The NPPA signal in the atrium (J and N) was used as a labelling control, as nppa is highly expressed in this tissue. (O) The cross-sectional area of WGA-labelled cardiomyocytes was compared showing no difference between CTRL 11mpa and SHAM 11mpa, whereas cell area was significantly increased for AMP 11mpa, revealing hypertrophic cardiomyocytes at the site of amputation. A slight increase was also seen between both CTRLs separated by 11 months, which suggested an aging effect. Count was performed on 2 or 3 [file pone.0173418.s004.tif]

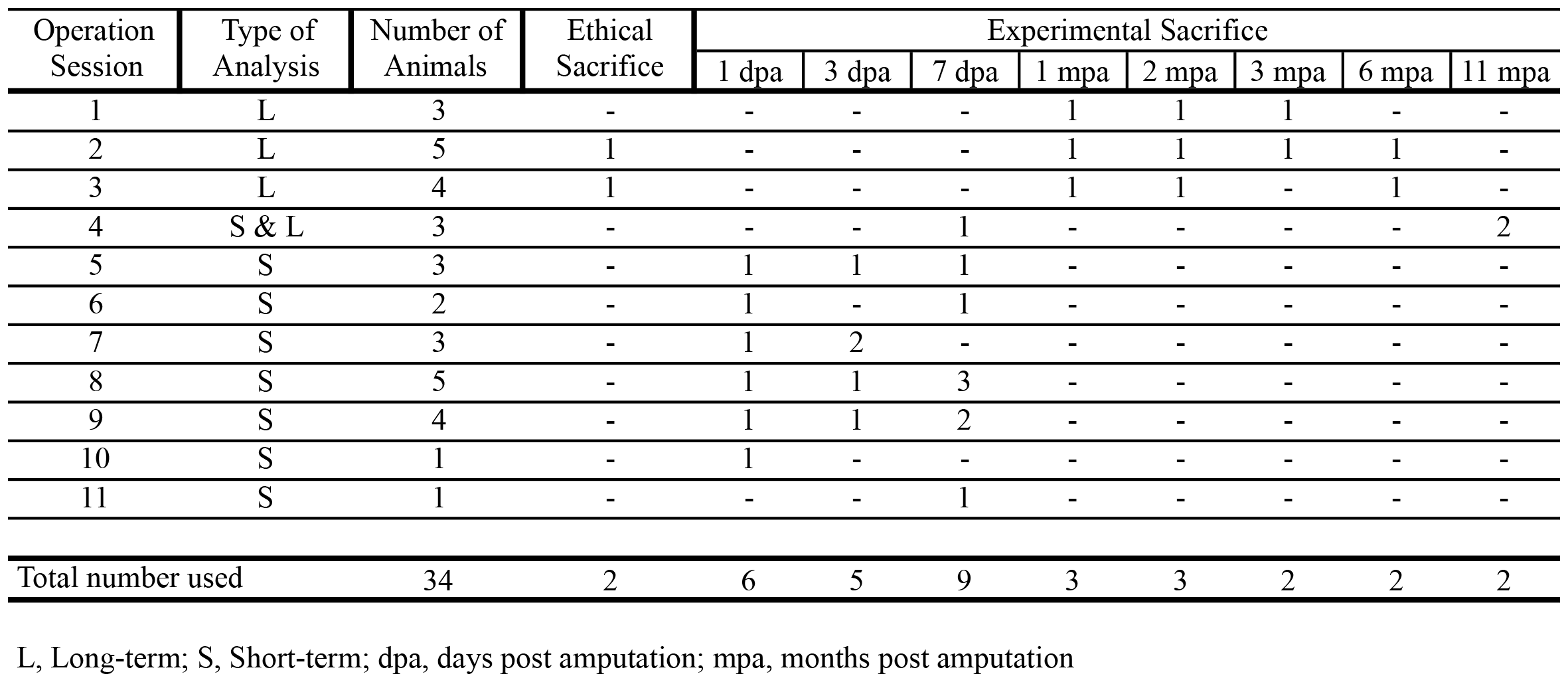

Supplement: S2 Table — The total number of adult Xenopus laevis that followed the complete endoscopic procedure and whether they were used for a long-term or short-term analysis. (TIF) [file pone.0173418.s006.tif]
