# Supplementary material for: Persistent fibrosis, hypertrophy and sarcomere disorganisation after endoscopy-guided heart resection in adult Xenopus
Source: PLoS One. 2017 Mar 9;12(3):e0173418. doi: 10.1371/journal.pone.0173418 (PMC5344503; doi:10.1371/journal.pone.0173418)
Supplement: S1 Table — For each primer pair, the forward and reverse sequences are given. Most genes studied were known as a single copy when this study was conducted except cebpb and ccnd1; these primers allowed the amplification of both cebpb homeologs but only a single ccnd1 homeolog. Target of Xenopus laevis long (.L) or short chromosome (.S) follows the gene name. (DOCX) [file pone.0173418.s005.docx]

Supplementary Table 1

| **Gene(s)** | **Forward (F) & Reverse (R) primer sequences** | **Gene accession number(s)** |
| --- | --- | --- |
| *smarcd1.L* (a.k.a *baf60*) | F: 5’-TGCAACAGGCTGCTCAGAATA-3’  R: 5’-TGGGATTCGGGTACCAGTTC-3’ | NM_001142786.1 |
| *smn2.L* | F: 5’-GAGACGCCAAAGTCCTCACA-3’  R: 5‘-AGCTTCTCACCATGCCTTCC-3’ | NM_001088917.1 |
| *actl6a.L* | F: 5’-ATTGAGCCACCAATCCAG G-3’  R: 5’-ACAAGTGTTGGAATGTGCG-3’ | NM_001086982.1 |
| *actn3.L* | F: 5’- GAGCTGAAGAAGGGGATTCCT-3’  R: 5’- CCTTCTATACCTCCTGCAGCC-3’ | NM_001093023.1 |
| *akt1.S* | F: 5’-GCTGGACAAAGACGGACACA-3’  R: 5’-CCCGCACATCATCTCGTACA-3’ | NM_001090409.1 |
| *ccnd1.L* | F: 5’-CACTTTTCCTATCACAGGTCATCAA-3’  R: 5’-TTCTTGGCACGCCCGTAA-3’ | NM_001086005.1 |
| *cebpb.L* and *cebpb.S* | F: 5’-AAGGGGGCGCTAGAGTACA-3’  R: 5’-CGCTCGGTACCGTTTGGTAA-3’ | NM_001095915.1  NM_001172167.1 |
| *col1a1.S* | F: 5’-TGGATACACGGACCCTGCT-3’  R: 5’-GTTTCCATTGTCGCACACACA-3’ | NM_001087352.1 |
| *ctgf.L* | F: 5’-TATCGATGGGGGTGTGGGAT-3’  R: 5’-CTGAAAGCAGGCAAAGCAGG-3’ | NM_001088228.1 |
| *cxcl8.L* (a.k.a *il8*) | F: 5’-TGGCAATACTGGCTCTCTGC-3’  R: 5’-TGGGGCCCTTTAGGGATCAT-3’ | NM_001097106.1 |
| *fn1.S* | F: 5’-GGCCCTGCTTTGTATCCCTT-3’  R: 5’-AATGGAACTGGGAGCAGACG-3’ | NM_001087801.1 |
| *il1b.S* | F: 5’-AAGCTGTCGTCCTTGTGGTT-3’  R: 5’-ATAGCGGTATGTGGAAGCGG-3’ | NM_001085605.1 |
| *nppa.L* | F: 5’-TGAGATTGGGAAAACACTCCTGA-3’  R: 5’-GCATGCTGTCCAAAGTCCTG-3’ | NM_001096918.1 |
| *nppb.L* | F: 5’-ACCCGTTGATGGACTTGGAC-3’  R: 5’-AGCCAGTTTCTCTTCCAGCC-3’ | NM_001095529.1 |
| *odc1.L* | F: 5’-TGAAAACATGGGTGCCTACA-3’  R: 5’-AAGTTCCATTCCGCTCTCCT-3’ | NM_001086698.1 |
| *pcna.L* | F: 5’-CGTCGCGGTAATCCCTTACA-3’  R: 5’-CCAACACCTTCTTCAGGATGGA-3’ | NM_001087542.1 |
| *tert.L* | F: 5’-CGGTCTCCTTTCTTCAGCTATCA-3’  R: 5’-CTCTTCTTTTCACAAATCCGTTGA-3’ | NM_001085633.1 |
| *tnnt2.L* | F: 5’-TGAAAGCCGGAAAAAGGAGGA-3’  R: 5’-CTTCTTTGCGTGCACGTTCT-3’ | NM_001089040.1 |

Table 1: Primer sequences used for Real-Time q-PCR experiments. For each primer pair, the forward and reverse sequences are given. Most genes studied were known as a single copy when this study was conducted except *cebpb* and *ccnd1*; these primers allowed the amplification of both *cebpb* homeologs but only a single *ccnd1* homeolog. Target of *Xenopus laevis* long (.L) or short chromosome (.S) follows the gene name.
